# Effects of exogenous hydrogen sulphide on calcium signalling, background (TASK) K channel activity and mitochondrial function in chemoreceptor cells

**DOI:** 10.1007/s00424-012-1089-8

**Published:** 2012-03-15

**Authors:** Keith J. Buckler

**Affiliations:** Department of Physiology Anatomy & Genetics, University of Oxford, Parks Road, Oxford, OX1 3PT UK

**Keywords:** Hydrogen sulphide, Calcium signalling, Chemoreceptors, Mitochondrial function, Oxygen sensing

## Abstract

It has been proposed that endogenous H_2_S mediates oxygen sensing in chemoreceptors; this study investigates the mechanisms by which H_2_S excites carotid body type 1 cells. H_2_S caused a rapid reversible increase in intracellular calcium with EC_50_ ≈ 6 μM. This [Ca^2+^]_i_ response was abolished in Ca-free Tyrode. In perforated patch current clamp recordings, H_2_S depolarised type 1 cells from −59 to −35 mV; this was accompanied by a robust increase in [Ca^2+^]_i_. Voltage clamping at the resting membrane potential abolished the H_2_S-induced rise in [Ca^2+^]_i_. H_2_S inhibited background K^+^ current in whole cell perforated patch and reduced background K^+^ channel activity in cell-attached patch recordings. It is concluded that H_2_S excites type 1 cells through the inhibition of background (TASK) potassium channels leading to membrane depolarisation and voltage-gated Ca^2+^ entry. These effects mimic those of hypoxia. H_2_S also inhibited mitochondrial function over a similar concentration range as assessed by NADH autofluorescence and measurement of intracellular magnesium (an index of decline in MgATP). Cyanide inhibited background K channels to a similar extent to H_2_S and prevented H_2_S exerting any further influence over channel activity. These data indicate that the effects of H_2_S on background K channels are a consequence of inhibition of oxidative phosphorylation. Whilst this does not preclude a role for endogenous H_2_S in oxygen sensing via the inhibition of cytochrome oxidase, the levels of H_2_S required raise questions as to the viability of such a mechanism.

## Introduction

It has been proposed that hydrogen sulphide is a naturally occurring gassotransmitter similar to nitric oxide [50]. It has also been hypothesised that endogenous H_2_S production plays a particularly important role in the process of acute oxygen sensing in blood vessels [36, 38], fish gill chemoreceptors [39] and the mammalian carotid body [37, 41]. Endogenous H_2_S is primarily produced through the metabolism of two sulphur-containing amino acids cysteine and homocysteine by the enzymes cystathionine β-synthase and cystathionine γ-lyase (γ-cystathionase) [23, 24, 26]. Its subsequent degradation is by oxidation to thiosulphate in the mitochondrion. This occurs in several stages; the first involves the formation of a persulphide with sulphide quinone oxidoreductase (SQR) together with reduction of ubiquinone; this is followed by oxidation of the persulphide by a sulphur dioxygenase to form sulphite; the final reaction is catalysed by a sulphur transferase which produces thiosulphate by transferring a second persulphide from SQR to sulphite [21]. This process requires oxygen for two functions, firstly to re-oxidise ubiqinone via the electron transport chain and secondly in the oxidation of persulphide by sulphur dioxygenase. In the H_2_S hypothesis, oxygen sensing is proposed to be initiated by alteration of the balance between constitutive H_2_S production and oxygen-dependent H_2_S removal [38].

The ability of exogenous H_2_S to act as a respiratory stimulant has long been recognised, possibly as far back as the 1800s [35]. An early contemporary account is given by Hagard and Henderson who, in 1922, reported that both inhalation of H_2_S gas and injection of 2 mg/kg Na_2_S evoked a hyperpnea in dogs [16]. The stimulatory effects of sulphides were subsequently traced to actions upon the carotid body [20]. The mechanisms by which H_2_S stimulates the carotid body are however largely undefined. It has been reported that chemoreceptor excitation by exogenous H_2_S can be blocked by removal of external calcium or application of cadmium [31, 41]. This suggests that H_2_S promotes Ca^2+^ influx as does hypoxia [6]. It has also been reported that H_2_S inhibits large conductance Ca^2+^-activated K^+^ channels (BK_Ca_) [31]. Inhibition of BK_Ca_ alone however is usually insufficient to excite chemoreceptor cells as these channels are inactive under resting conditions [4]. Excitatory responses to hypoxia are primarily mediated by the inhibition of a background potassium current believed to be carried through TWIK-related acid*-*sensitive potassium (TASK) channels [4, 10, 25]. The effects of H_2_S on these channels are unknown.

There is also uncertainty in the nature of the signalling pathways linking hydrogen sulphide to modulation of ion channel activity. In blood vessels, H_2_S has been reported to directly activate K_ATP_ channels leading to membrane hyperpolarisation, reduction of voltage-gated Ca^2+^ influx and vasodilation [56]. H_2_S has also been reported to inhibit large conductance Ca^2+^-activated K^+^ channels (BK_Ca_) in excised patches [47], again suggesting direct interaction between H_2_S and the ion channel. H_2_S is however also a powerful inhibitor of cytochrome oxidase [13]. Indeed, a general criticism of the hypothesised role for H_2_S as a gasotransmitter is that the majority of studies demonstrating effects of exogenous H_2_S have employed concentrations that have the potential to poison energy metabolism. This issue is of particular importance when considering a potential role for H_2_S in oxygen sensing in peripheral chemoreceptors since these organs are sensitive to many inhibitors of oxidative phosphorylation [2, 8, 11, 20, 34, 44, 54]. This study therefore seeks to address two main issues in relation to the actions of H_2_S: (1) how does H_2_S excite the type 1 cell and are these mechanisms the same as those observed in hypoxia and (2) can the effects of H_2_S be attributed to a novel signalling pathway or are they simply due to metabolic inhibition.

## Methods

### Type 1 cell isolation

Carotid bodies were excised from neonatal rat pups (11–15 days) under terminal anaesthesia (2–4% halothane) in accordance with project and personal licences issued under the UK Animals (Scientific Procedures) Act 1986. Type 1 cells were isolated using enzymatic digestion with 0.3 mg/ml trypsin (Sigma) and 0.5 mg/ml collagenase (Worthington) in PBS or Ham’s F-12 for 25–30 min at 35°C followed by transfer to enzyme-free culture media (see below) and trituration through fire-polished pipettes. The resultant cell suspension was plated onto poly-l-lysine-coated coverslips and maintained in an incubator at 35°C for 2 h before the addition of further culture media. Cells were used within 8 h of isolation. Culture media comprised Ham’s F-12 or DMEM containing 10% heat-inactivated foetal bovine serum, 2 mM l-glutamine and 4 μg/ml insulin.

### Measurement of [Ca^2+^]_i_, [Mg^2+^]_i_ and NADH

Fluorescence measurements were performed using a microspectrofluorimeter based on a Nikon Diaphot 200 (Japan) equipped with a xenon lamp to provide an excitation light source and cooled (−20°C) photomultiplier tubes (Thorn EMI) to detect emitted fluorescence. [Ca^2+^]_i_ was determined using Indo-1, [Mg^2+^]_i_ using Mag-Indo-1 and NADH by cellular autofluorescence. Indo-1 and Mag-Indo-1 were loaded into cells by incubation with 2–5 μM of the acetoxymethyl ester derivatives of these dyes in culture media at room temperature for 1 h (Indo-1) or 10 min (Mag-Indo-1). Indo-1 and Mag-Indo-1 were excited at 340 nm and fluorescence intensity measured at 405 ± 16 and 495 ± 10 nm. The fluorescence emission ratio (405/495) for Indo-1 was calibrated as previously described [5]; the fluorescence ratio 405/495 for Mag-Indo-1 is presented without calibration. NADH autofluorescence was excited at 340 nm and emission measured at 450 ± 30 nm. Data acquisition and analysis was performed using a 1401 interface and Spike 2 software (Cambridge Electronic Design).

### Electrophysiology

Both perforated patch and cell-attached patch recordings were performed using an Axopatch 200B. Electrodes were pulled from borosilicate glass tubing and were sylgarded and fire polished just before use. For perforated patch recording, pipette filling solutions contained (in millimolars) K_2_SO_4_ 70, potassium chloride (KCl) 30, ethylene glycol tetraacetic acid (EGTA) 1, (4-(2-hydroxyethyl)-1-piperazineethanesulfonic acid ) (HEPES) 10 and MgCl_2_ 2, and pH was adjusted to 7.2 at 37°C. Amphotericin was added to this solution from a stock solution in DMSO to a final concentration of 120–240 μg/ml just prior to recording. For cell- attached patch recording, pipette filling solutions contained (in millimolars) KCl 140, MgCl_2_ 1, EGTA 1, HEPES 10, tetraethylammonium 10, 4-aminopyridine 5. pH was adjusted to 7.4 at 37°C.

Single-channel recordings were performed at a filter frequency of 2 kHz, and membrane current was digitised and recorded at 5–20 kHz. Membrane current and voltage during perforated patch recordings were sampled at 5 kHz. Data acquisition, voltage clamp control and data analysis were performed using Spike 2 software. Single-channel activity was quantified as NPopen using the main conductance state to set a 50% opening threshold. Current levels of greater than 150% of the main conductance state were counted as multiple openings.

Perforated patch recordings were performed with simultaneous measurement of [Ca^2+^]_i_. Cells failing to maintain low resting levels of [Ca^2+^]_i_ following patch formation and perforation were rejected (see [4]). Whole-cell current–voltage (I/V) relationships were determined by using voltage ramps. In these experiments, average I/V curves (as in Fig. 4c) were constructed by averaging digitised current and voltage data first over 0.5-mV intervals, then over 5–15 successive ramps and finally I/Vs from individual cells were averaged.

It was noted that H_2_S frequently caused an offset at the reference electrode of between 3 and 10 mV. Raw recordings of membrane potential (i.e. Fig. 3a, b) are presented without correction for this offset, but the offset has been corrected for in the summary data presented in the text and Fig. 3c. This error has also been corrected for in recordings of membrane current and current–voltage relationships (Fig. 4). In single-channel recordings, a second potential source of voltage offset may result from depolarisation of the resting membrane potential in response to H_2_S or CN^−^. In these experiments, no explicit correction for change in membrane voltage (or voltage offset) was made (as these channels are voltage insensitive), but single-channel amplitude was determined under each condition from all-points histograms so that thresholds for determining NPopen could be adjusted to take into account any changes in membrane voltage.

### Solutions

Standard bicarbonate-buffered Tyrode solutions contained (in millimolars): NaCl 117, KCl 4.5, CaCl_2_ 2.5, MgCl_2_ 1, NaHCO_3_ 23 and Glucose 11. In Ca^2+^-free solutions, CaCl_2_ was omitted and 100 μM EGTA was added. Twenty millimolars K^+^ Tyrode contained 20 mM KCl and 101.5 mM NaCl, all other constituents remained the same. High-K^+^ low-Ca^2+^ solutions for cell-attached patch recordings contained (in millimolars) NaCl 21.5, KCl 100, MgCl_2_ 1, NaHCO_3_ 23 and Glucose 11. Normoxic solutions were equilibrated 5% CO_2_ and 95% air, hypoxic solutions were equilibrated with 5% CO_2_ and 95% N_2_ (*P*_O2_ = 2 Torr); both had a pH of 7.4 at 37°C. Note that for solutions containing CN^−^ or NaHS, Tyrodes were bubbled for >15 min prior to the addition of these compounds and thereafter maintained under an atmosphere of 5% CO_2_/95% air (i.e. these solutions were not continuously bubbled as both compounds are volatile in their acid form and would be rapidly lost from solution). NaCN and NaHS were added from stock solutions freshly prepared just before use. H_2_S concentration was estimated assuming pK_a_ = 6.9 [46]. Note that different conventions are used in reporting H_2_S levels; some authors present the amount of the sulphide salt added to solution rather than the resulting concentration of H_2_S. For comparison, in this study, the total NaHS added to solution is approximately four times the quoted H_2_S concentration. Different values for pK_a_ have also been used, e.g. Whitfield et al. [51] employed a pKa of 6.6 for mammalian blood at 37°C; applying this value for pKa would give H_2_S concentrations approximately 4/7^ths^ (57%) of those reported here.

### Statistical analysis of data

Values are expressed as mean±standard error of mean. Most comparisons were assessed using a two-tailed *t* test (paired or unpaired as appropriate). Dose–response data for the effects of H_2_S on intracellular calcium, magnesium and NADH fluorescence were analysed using one-way repeated measures ANOVA or Friedman repeated measures ANOVA on ranks. This was followed by multiple comparisons versus control using the Holm–Sidak method or Dunnett’s method, respectively. Standard curves were obtained by fitting the four-parameter logistic curve

$$ {\text{Response }} = { \min } + {\text{ (max}} - {\text{min)}}/({1 } + { ([}{{\text{H}}_{{2}}}{\text{S]}}/{\text{EC5}}0{{)}^n}){\text{ where }}n = {\text{ Hill coefficient}} $$

Significance was assumed at *p* < 0.05 for *t* tests, ANOVA and post hoc comparisons against control using Dunnett’s method. For post hoc comparisons using the Holm–Sidak method, values were considered significant when *p* < the critical level, starting at *p* < 0.05. Statistical analysis and curve fitting were carried out using SigmaPlot 11 (Systat Software Inc, Germany) or Excel (Microsoft).

### Drugs

NaSH and EGTA were from Sigma. Indo-1-AM and Mag-Indo-1-AM were from Molecular Probes.

## Results

### Effects of exogenous H_2_S on intracellular Ca^2+^ signalling

**Fig. 1:**
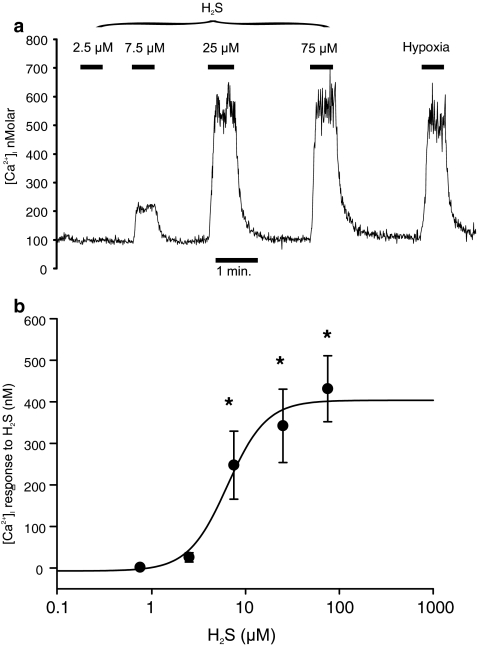
Effects of H_2_S on [Ca^2+^]_i_ in type 1 cells. [Ca^2+^]_i_ responses to application of graded levels of H_2_S from 2.5–75 μM. **a** Example of a recording demonstrating rapid rise in [Ca^2+^]_i_ in response to brief application of H_2_S at concentrations of 7.5 μM and above. Response to a hypoxic stimulus (PO_2_ = 2 Torr) is also shown for comparison. **b** Dose–response relationship for H_2_S-mediated increase in [Ca^2+^]_i_. Curve is best fit to a four-parameter logistic equation (see “Methods” section) with EC_50_ = 6 μM. *p < 0.05 (Dunnett’s method), *n* = 8

Exogenously applied H_2_S caused an abrupt increase in intracellular calcium (*p* < 0.001; Friedman repeated measures ANOVA on ranks) from concentrations of 7.5 μM and above (see Fig. 1). This [Ca^2+^]_i_ response rapidly reversed upon removal of H_2_S. The dose-dependent effects of H_2_S on [Ca^2+^]_i_ were well described by a four-parameter logistic curve (see “Methods” section) with an EC_50_ of approx 6 μM (Fig. 1b).

**Fig. 2:**
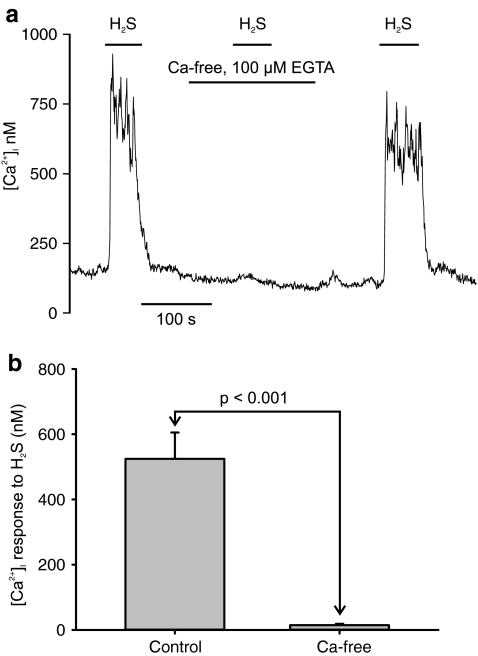
H_2_S promotes Ca^2+^ influx. **a** Effects of 25 μM H_2_S on [Ca^2+^]_i_ in presence and absence of extracellular Ca^2+^. Ca^2+^-free solutions also contained 100 μM EGTA. **b** Summary of effects of Ca^2+^-free media (+EGTA) on [Ca^2+^]_i_ response to 25 μM H_2_S. Response was measured as [Ca^2+^]_i_ during exposure to H_2_S–[Ca^2+^]_i_ prior to exposure to H_2_S. *p* value was determined by paired *t* test (two tailed), *n* = 7

In order to determine whether the H_2_S-mediated rise in [Ca^2+^]_i_ was due to Ca^2+^ influx or Ca^2+^ release from internal stores, the effects of 25 μM H_2_S were studied in both a normal Tyrode and a Ca-free Tyrode containing 100 μM EGTA. In the Ca-free Tyrode, the response to H_2_S was, on average, inhibited by >95% (Fig. 2). The mean rise in [Ca^2+^]_i_ in control Tyrode was 524 ± 81 nM, whereas that in Ca-free Tyrode was 15 ± 4 nM. This difference was highly significant (*p* < 0.001, *n* = 7) and provides compelling evidence that H_2_S causes a substantial Ca influx in type 1 cells.

### Effects of exogenous H_2_S on membrane potential and background K currents

**Fig. 3:**
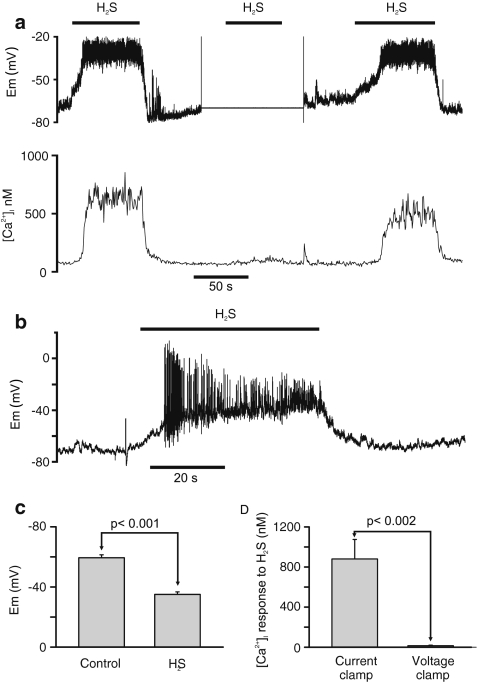
H_2_S promotes membrane depolarisation and voltage-gated Ca^2+^ entry. **a** Simultaneous recording of membrane potential, using the perforated patch technique, and intracellular calcium in a single type 1 cell. Application of 25 μM H_2_S causes a rise in [Ca^2+^]_i_ coincident with a rapid membrane depolarisation in current clamp (*I* = 0) but little change in [Ca^2+^]_i_ when the cell is voltage clamped at its resting membrane potential. **b** Recording of membrane depolarisation in another type 1 cell during exposure to 25 μM H_2_S showing action potential generation. **c** Summary of effects of 25 μM H_2_S on average membrane potential in current clamped type 1 cells (*n* = 11). **d** Summary of effects of voltage clamp on [Ca^2+^]_i_ response to 25 μM H_2_S. The [Ca^2+^]_i_ response was calculated as the difference between baseline [Ca^2+^]_i_ prior to and [Ca^2+^]_i_ during exposure to H_2_S under both current clamp and voltage clamp conditions (*n* = 11). *p* values were determined by paired *t* test

Type 1 cells also respond to natural chemostimuli including hypoxia and acidosis with Ca influx which serves to stimulate neurosecretion and thus excitation of afferent nerves. In both of these circumstances, the [Ca^2+^]_i_ response is mediated by voltage-gated Ca entry in response to membrane depolarisation and, in many cells, the initiation of repetitive action potentials [6, 7]. The effects of H_2_S upon membrane potential were therefore investigated using perforated patch whole cell recording. In this series of experiments, the resting membrane potential of type 1 cells was −59 ± 2.0 mV (*n* = 11) under current clamp recording conditions (Im = 0). Following exposure to 25 μM H_2_S, all cells depolarised by, on average, approximately 24 mV to a mean potential of −35 ± 1.7 mV (*n* = 11, *p* < 0.001, Fig. 3a–c). In most cells, this was accompanied by weak electrical activity (e.g. Fig. 3a), although a few cells fired more robust action potentials (e.g. Fig. 3b). This depolarisation was typically rapid in onset (i.e. within 10 s of application of H_2_S) and cells repolarised rapidly following H_2_S removal. In all instances, H_2_S-induced membrane depolarisation was accompanied by a robust increase in [Ca^2+^]_i_ from a mean resting level of 109 ± 14 to 990 ± 195 nM in the presence of H_2_S (*n* = 11, *p* < 0.002). Cells were then placed into voltage clamp with membrane potential held close to the cells’ normal resting membrane potential (e.g. Fig. 3a). Under voltage clamp conditions, mean resting [Ca^2+^]_i_ was 124 ± 18 nM. With membrane potential thus prevented from changing, H_2_S was applied for a second time during which [Ca^2+^]_i_ rose only very slightly to 139 ± 17 nM. Voltage clamping the cell thus inhibited the H_2_S-induced rise in [Ca^2+^]_i_ by, on average, 98% (*p* < 0.002, *n* = 11, see Fig. 3d). These experiments confirm that the principal cause of the H_2_S-induced rise in [Ca^2+^]_i_ is membrane depolarisation-driven voltage-gated Ca^2+^ entry as is the case for other chemostimuli (see “Discussion” section).

### Effects of H_2_S on background K^+^ currents and channels

**Fig. 4:**
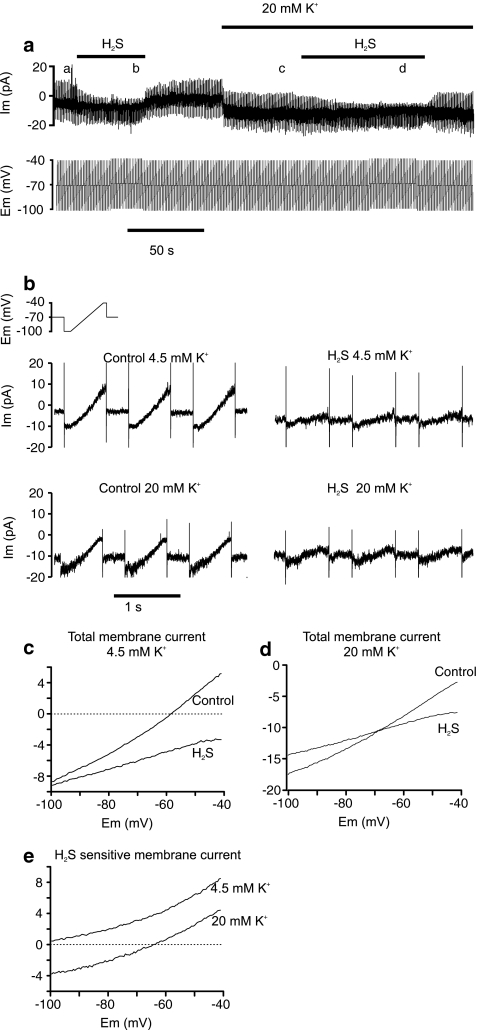
H_2_S inhibits background K^+^ current in type 1 cells. **a** Perforated patch whole cell voltage clamp recording of membrane current and membrane potential in a type 1 cell exposed to 25 μM H_2_S in both normal Tyrode (4.5 mM [K^+^]_o_) and high potassium Tyrode (20 mM [K^+^]_o_). Note small offset in voltage clamp protocol in presence of H_2_S to correct for the effects of H_2_S on the reference electrode (see “Methods” section). **b** *Top left* trace shows the voltage clamp ramp protocol on a faster time base. *Middle* and *lower* traces show membrane current recorded over 3-s intervals in control Tyrode, control Tyrode + 25 μM H_2_S, 20 mM K^+^ Tyrode and 20 mM K^+^ Tyrode + 25 μM H_2_S. These recordings were taken at points *a*, *b*, *c* and *d* during the recording shown in panel **a**. Note marked reduction in ramp current during the application of H_2_S. **c** Average current–voltage relationships obtained from ramp protocols in normal Tyrode with and without H_2_S (*n* = 7 cells). **d** Average current–voltage relationships obtained from ramp protocols in 20 mM K^+^ Tyrode with and without H_2_S (*n* = 7 cells). **e** H_2_S-sensitive current recorded in normal Tyrode and high (20 mM) K^+^ Tyrode. H_2_S-sensitive current was determined by subtraction of membrane current recorded in the presence of H_2_S from that recorded in its absence (control). Note the marked shift in the current–voltage relationship with elevated K^+^ and a reversal potential in 20 mM K^+^ close to the predicted value for *E*_K_ (=−56 mV)

In order to determine the cause of the H_2_S-induced depolarisation, cells were voltage clamped to −70 mV and subjected to repeated voltage ramps from −100 to −40 mV at 1 Hz (see Fig. 4a, b). Application of 25 μM H_2_S caused a marked reduction in the membrane current generated in response to these voltage ramps, see Fig. 4a and compare left and right hand panels of Fig. 4b. Figure 4c shows averaged current–voltage relationships (I/V) for seven cells in normal Tyrode in the presence and absence of H_2_S. The point at which the control I/V transects the 0 current axis represents the resting membrane potential; note that there is no stable resting membrane potential in this voltage range in the presence of H_2_S (consistent with the membrane depolarisation recorded in current clamp, see above). The slope of the I/V relationship is also notably less steep in the presence of H_2_S than under control conditions, indicating that H_2_S reduces resting membrane conductance. H_2_S reduced membrane conductance, measured between −55 and −65 mV by approximately 60% from 278 ± 26 pS under control conditions to 106 ± 22 pS (*p* < 0.005). Cells were then superfused with a high (20 mM) K^+^ Tyrode and a further set of voltage clamp measurements taken under both control conditions and in the presence of 25 μM H_2_S (see Fig. 4a,b and d). H_2_S also reduced membrane conductance in the high K tyrode (Fig. 4d). The current–voltage relationship of H_2_S-sensitive current was then calculated both in normal Tyrode and in high K Tyrode by subtracting the current obtained in the presence of H_2_S from that obtained under control conditions. This H_2_S-sensitive current is displayed in Fig. 4e. Note that the H_2_S-sensitive current is displaced downward in elevated external potassium. Under control conditions, although the average I/V (from all seven cells) did not show a reversal potential within the voltage range tested, a reversal potential was observed in four cells (between −83 and −97 mV, mean −90 ± 3 mV). In the presence of 20 mM external K^+^, all seven cells showed a reversal potential (mean −65 ± 6 mV). These reversal potentials are close to the equilibrium potential for potassium ions under these recording conditions (−94 and −56 mV for normal and high K Tyrode, respectively). These data indicate that H_2_S inhibits a background/resting potassium conductance and that this is probably the primary factor in causing membrane depolarisation.

**Fig. 5:**
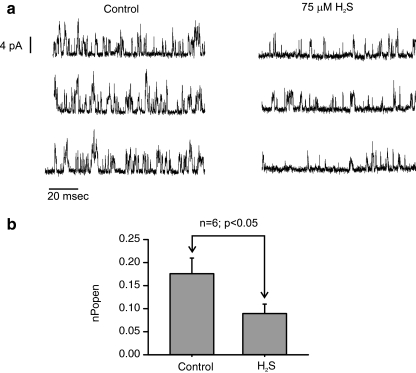
Effects of H_2_S on background K^+^ channels. **a** Cell-attached patch recording of background K^+^ channel activity in a type 1 cell. *Upward* deflections show channel opening and current flow into the cell (inward current). Cells were bathed in a 100-mM K^+^ Tyrode (see “Methods” section). Pipette solution contained 140 mM K^+^. Pipette potential was +80 mV. Traces on the *left* are sections of recording obtained under control conditions, those on the *right* were obtained in the presence of 75 μM H_2_S. **b** Comparison of background K^+^ channel activity in cell-attached patches (as above) under control conditions and in the presence of 75 μM H_2_S. *n* = 6 cells; *p* value determined by paired *t* test

To confirm the effects of H_2_S on background K^+^ channels, cell-attached patch recordings were performed. As previously described, by this lab and others, under appropriate recording conditions, there is abundant single-channel activity in these cells at the resting membrane potential from voltage-insensitive background K channels which appear to be comprised of members of the TASK-1/TASK-3 channel family, probably predominantly TASK1/3 heteromers [10, 25, 52]. Channel activity (Fig. 5) was recorded at a pipette potential of +80 mV with the cell bathed in a 100 mM K^+^ and Ca-free Tyrode and with a pipette filling solution containing 140 mM K^+^ and 1 mM Mg^2+^ (see “Methods” section for full composition). Under these recording conditions, the mean current amplitude of the main conductance state as determined from all-points histograms was 3.4 ± 0.1 pA (note that the conductance of these channels is dependent upon extracellular Mg^2+^ concentration; see [52]). The main conductance state was used to set a 50% threshold for the determination of NPopen. Single-channel activity was approximately halved by 75 μM H_2_S from 0.18 ± 0.03 to 0.09 ± 0.02 (*n* = 6; *p* < 0.05) see Fig. 5.

### Effects of H_2_S on mitochondrial function

The effects of H_2_S described above are identical to those of hypoxia (see “Discussion” section); they are also identical to those of many inhibitors of mitochondrial respiration [8, 54]. Since H_2_S is a well-known inhibitor of cytochrome oxidase [13], this poses the question as to whether the effects of H_2_S are mediated primarily through the inhibition of oxidative phosphorylation or by some other means. The effects of H_2_S on mitochondrial function were therefore investigated.

**Fig. 6:**
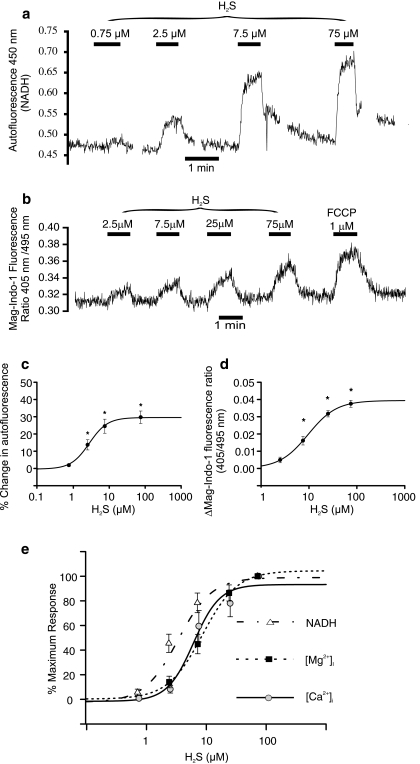
Effects of H_2_S on mitochondrial metabolism. **a** Measurement of cellular autofluorescence (excitation 340 nM, emission 450 nM) as an index of NADH levels. *Upward* deflection is consistent with increased NADH. Cell was bathed in normal Tyrode and subject to repeated 1-min exposures to H_2_S in increasing concentrations. Note rapid increase in fluorescence/NADH at higher levels of H_2_S. **b** Measurement of free [Mg^2+^]_i_ using Mag-Indo-1. Increase in fluorescence ratio 405/495 denotes increase in free Mg^2+^. Increase in [Mg^2+^]_i_ indicates MgATP hydrolysis (see text). **c** Dose–response plot for effects of H_2_S on autofluorescence. Curve represents four-parameter logistic equation with an EC_50_ of 2.8 μM. *Asterisks* indicate data points significantly different from control (using Holm–Sidak method, see “Methods” section). **d** Dose–response plot for effects of H_2_S on [Mg^2+^]_i_ (Mag-Indo-1 fluorescence ratio 405/495). Curve represents four-parameter logistic equation with an EC_50_ of 9.7 μM. *Asterisks* indicate data points significantly different from control (using Holm–Sidak method). **e** Comparison of dose–response data for effects of H_2_S on [Ca^2+^]_i_ , [Mg^2+^]_i_ and NADH. All data were normalised on a 0 (control) to 100% (maximum response) scale and curves recalculated using four-parameter logistic equations. Data points at 75 μM are superimposed. Note proximity of both [Ca^2+^]_i_ and [Mg^2+^]_i_ responses with the NADH response displaced slightly to the *left* (lower H_2_S concentrations)

Inhibition of the electron transport chain results in the accumulation of mitochondrial NADH. Since NADH is fluorescent (and NAD is not), changes in NADH levels can be observed by recording cellular autofluorescence (see “Methods” section and [12]). Figure 6a shows a typical recording of type 1 cell autofluorescence and the effects of varying levels of H_2_S upon it. H_2_S causes a rapid and reversible increase in cellular autofluorescence (*p* < 0.001, *n* = 7). This effect was significant at concentrations of 2.5 μM and above. The dose–response relation was well described by a four-parameter logistic curve (see “Methods” section) with an EC_50_ of 2.8 μM (Fig. 6c).

Inhibition of oxidative phosphorylation can also lead to a decline in cellular ATP levels. Since ATP is a chelator of intracellular Mg^+^, a decline in ATP levels results in the release of Mg^2+^ into the cytosol. Under normal conditions, cellular levels of Mg^2+^ are relatively low, e.g. 0.3–1.25 mM [18, 32, 45], and although [Mg^2+^ ]_i_ is actively regulated, transmembrane fluxes are invariably slow [15, 42, 43]. As a consequence, resting [Mg^2+^]_i_ tends to be relatively constant. For the above levels of free Mg, most ATP within the cell would be expected to be complexed with Mg^2+^, depletion of ATP by conversion first to ADP and then to AMP (by adenylate kinase) is therefore associated with the release of significant amounts of Mg^2+^ and a readily detectable rise in cytosolic [Mg^2+^]_i_ [14, 19, 30]. Thus, ATP depletion can be followed by measuring [Mg^2+^]_i_. Rapid elevation of [Mg^2+^]_i_ has previously been reported to occur in type 1 cells following the application of many other inhibitors of oxidative phosphorylation including cyanide, rotenone, oligomycin and 2-4-dinitrophenol [48]. Figure 6b shows a recording of [Mg^2+^]_i_ in a type 1 cell using Mag-Indo-1. H_2_S causes a reversible increase in [Mg^2+^]_i_ (*p* < 0.001, *n* = 9) as did another inhibitor of mitochondrial energy metabolism 1 μM FCCP (Fig. 6b). This effect of H_2_S was dose dependent; the rise in [Mg^2+^]_i_ was significant at H_2_S concentrations of ≥7.5 μM, and the dose–response relationship was well described by a four-parameter logistic curve with an EC_50_ of 9.7 μM (Fig. 6d).

A comparison of the concentration-dependent effects of H_2_S on [Ca^2+^]_i_, NADH levels and [Mg^2+^]_i_ is presented in Fig. 6e (with each dose–response curve normalised to a 0–100% scale). It is apparent from these data that the dose dependency of the effects of H_2_S upon type 1 cell [Ca^2+^]_i_ is comparable to the dose-dependent effects of H_2_S on mitochondrial function in these cells.

### Lack of additive effect of H_2_S and other electron transport inhibitors on K_B_ (TASK) channel activity

**Fig. 7:**
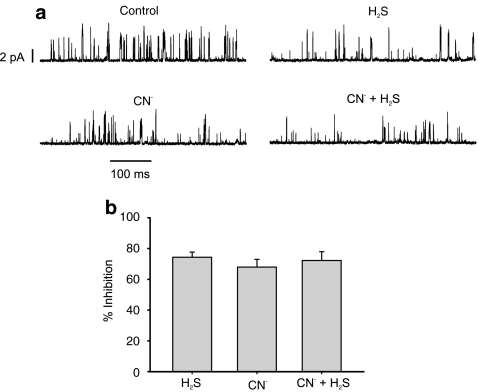
Effects of cyanide and H_2_S on background K channel activity. **a** Short sections of a cell-attached patch recording of background K^+^ channel activity in a type 1 cell. *Upward* deflections show channel opening and current flow into the cell (inward current). Cells were bathed in a 100-mM K^+^ Tyrode (see “Methods” section). Pipette solution contained 140 mM K^+^. Pipette potential was +80 mV. Traces show channel activity under control conditions, in the presence of 2 mM CN^−^, in the presence of 75 μM H_2_S and in the presence of both 2 mM CN^−^ and 75 μM H_2_S. **b** Comparison of inhibitory effects of 75 μM H_2_S, 2 mM CN^−^ and 75 μM H_2_S plus 2 mM CN^−^ on channel activity. Data are expressed as percent inhibition of NPopen in the presence of CN^−^ and H_2_S relative to control; *n* = 8 cells/patches

Given the similarity of the dose dependency of inhibition of mitochondrial function with that of excitation of the type 1 cell, and the previously well-described sensitivity of this tissue to excitation by other inhibitors of oxidative phosphorylation, the question arises as to whether H_2_S can influence channel activity by any mechanism other than inhibition of mitochondrial function. To address this issue, the effects of H_2_S were compared with those of another classical inhibitor of cytochrome oxidase, cyanide in single-channel recordings from the same cell-attached patches. Both compounds, at saturating levels (2 mM CN^−^ [54], 75 μM H_2_S), caused a marked reduction in background (TASK) channel activity in cell-attached patches (Fig. 7a). The degree to which H_2_S and CN^−^ reduced channel open probability (NPopen, see “Methods” section) was similar at 74 ± 3.4% and 68 ± 5% (difference not significant). Combination of H_2_S and CN^−^ also reduced NPopen by 72 ± 5.7% which was not significantly different from the effects of CN alone (Fig. 7b, *n* = 8 patches). H_2_S therefore had no additional effect on channel activity over that of cyanide.

## Discussion

### Effects of H_2_S on Ca^2+^ signalling in type 1 cells

Application of sulphide evoked an elevation of cytosolic calcium in all oxygen-sensitive type 1 cells studied. This effect was rapid in onset and also reversed rapidly upon removal of H_2_S. The EC_50_ for the H_2_S-evoked rise in [Ca^2+^]_i_ was estimated to be about 6 μM H_2_S. This calcium response was abolished by removal of extracellular calcium. These effects are comparable to the actions of sulphides on carotid sinus nerve (CSN) activity in isolated rat carotid body which is rapidly and reversibly excited by 30 μM NaHS (approximately 7.5 μM H_2_S) and abolished by [Ca^2+^]_o_ removal [41]. The effects of sulphide on CSN discharge rate are also abolished by a combination of P2X and nicotinic receptor antagonists [31]. Collectively, these data argue that the principal site of action of sulphide within the carotid body is presynaptic, i.e. at the type 1 cell, and that calcium signalling plays a prominent role in promoting this response.

### Mechanisms of H_2_S signalling and their similarity to hypoxia

The effects of hypoxia on isolated rat type 1 cells include inhibition of background potassium channels (K_B_) [4], which are thought to be derived from TASK1 and TASK3 with a possible preponderance of the heterodimeric form TASK1–TASK3 [10, 25, 52]), and inhibition of calcium-activated large-conductance potassium channels (BK_Ca_) [40]. The inhibition of K_B_ (TASK) channels leads to membrane depolarisation since these channels provide the major resting potassium conductance which maintains the negative resting membrane potential. Upon K_B_ channel inhibition other, as yet poorly defined, background inward currents depolarise the cell until the threshold for activation of voltage-gated calcium channels is reached. At this point, Ca^2+^ floods into the cell, action potentials may be generated and other voltage-gated and Ca^2+^-activated channels may become active.

The effect of exogenous H_2_S, as described in this paper, is directly comparable to those described above for hypoxia, i.e. inhibition of K_B_ (TASK) channels causing membrane depolarisation, electrical activity and voltage-gated Ca entry. Like hypoxia, H_2_S has also been reported to inhibit BK_Ca_ [47], although the inhibition of BK_Ca_ seems to require much higher levels of H_2_S than those needed to excite intact cells and tissues. In all major respects, therefore, exogenous H_2_S mimics the effects of hypoxia. It is however by no means unique in this regard, other inhibitors of cytochrome oxidase including cyanide, azide and carbon monoxide similarly mimic the effects of hypoxia [2, 3, 20, 22, 53, 54].

### Effects of H_2_S on mitochondrial function

The similarity between the effects of H_2_S, hypoxia and other complex IV inhibitors raises the question as to whether the effects of H_2_S can simply be attributed to inhibition of complex IV. To address this issue, it is pertinent to consider whether H_2_S actually alters mitochondrial function over the range of concentrations for which it acts as a chemostimulant. Direct measurement of mitochondrial function in this tissue is not practical due to its small size; consequently, two indirect methods have been employed.

The first method measured NADH levels by its intrinsic fluorescence (NAD is not fluorescent). In short-term experiments, increase in autofluorescence indicates increase in NADH/NAD ratio which may arise from impaired NADH oxidation. The data (Fig. 6) show that H_2_S is able to elevate NADH levels from quite low concentrations. There are, however, two distinct mechanisms by which H_2_S could increase NADH. At low concentrations, H_2_S is oxidised by mitochondrial sulphide quinione reductase (SQR) with concomitant reduction of ubiquinone [28]. The reduced ubiquinone is then oxidised by complex III of the electron transport chain. The oxidation of H_2_S (by SQR) could in principle compete with complex I for ubiquinone [28] if the flux of electrons through SQR is sufficient. Thus, low levels of H_2_S might cause a small increase in NADH without compromising oxidative phosphorylation. At higher levels, however, the H_2_S-induced rise in NADH is most likely to be due to inhibition of electron transport.

The second method evaluated the effects of H_2_S upon oxidative phosphorylation. This employs the measurement of free Mg^2+^ concentration within cells. Much of the Mg^2+^ within cells is bound to ATP, such that whenever there is net MgATP hydrolysis free Mg^2+^ is released into the cytosol and [Mg^2+^]_i_ increases (see e.g. [1, 19, 30]). These experiments were conducted in a Ca-free Tyrode to prevent Ca^2+^ influx during exposure to H_2_S and possible interference with the Mag-Indo1 signal. The rise in [Ca^2+^]_i_ encountered under Ca_o_^2+^-free conditions (15 nM) is not expected to have any measureable effect on Mag-Indo fluorescence which has a Kd for Ca^2+^ of around 34 μM [29]. As H_2_S is a weak acid, it could also influence intracellular pH. The sensitivity of Mag-indo-1 to pH interference is however negligible between 7.2 and 7.0 [19], and the concentrations of H_2_S employed even at the highest level (75 μM H_2_S, 300 μM total sulphide) are unlikely to cause an intracellular acidification of more than about 0.003 pH units (assuming a total intrinsic [9] plus open system HCO_3_^−^/CO_2_ buffering capacity of 45 mM/pH at pH_i_ = 7.2).

Measurements of [Mg^2+^]_i_ indicate that the dose–response curve for the effects of H_2_S on MgATP is right shifted compared to the effects of H_2_S on NADH levels (EC50 = 2.8 μM for NADH and 9.7 μM for [Mg^2+^]_i_). These data are compatible with the hypothesis that H_2_S may serve as a substrate for electron transport at low levels and an inhibitor of electron transport and oxidative phosphorylation at higher levels. It may also simply reflect a greater sensitivity of NADH levels to inhibition of cytochrome oxidase activity.

Comparison of the effects of H_2_S on [Ca^2+^]_i_ with the above two indices of mitochondrial function revealed a particularly close correlation with the effects of H_2_S on [Mg^2+^]_i_ indicating that the effects of H_2_S on [Ca^2+^]_i_ may be linked to the decline in MgATP. The observations that the effects of H_2_S on channel activity are similar to those of cyanide and that H_2_S has no additional effect upon K_B_ channel activity in the presence of cyanide (Fig. 7) support the conclusion that the actions of H_2_S are primarily mediated via inhibition of oxidative phosphorylation. The mechanisms by which mitochondrial energy metabolism regulate K_B_ channel function in type 1 cells have not yet been fully resolved, but two candidate pathways have been proposed; these are (1) direct modulation of the TASK channels by changes in MgATP levels [48] and (2) indirect modulation via changes in AMP/ATP levels and an AMP kinase [55]. Some questions have however been raised as to whether TASK channels can be directly modulated by AMP kinase [27].

In conclusion, the excitation of peripheral chemoreceptors by H_2_S can be explained by H_2_S’s ability to inhibit cytochrome oxidase and the type 1 cells widely reported sensitivity to anything that disrupts oxidative phosphorylation. There was no evidence for any significant involvement of non-mitochondrial signalling pathways in regulating K_B_ channels or of effects of H_2_S on [Ca^2+^]_i_ at concentrations less than those which interfere with mitochondrial metabolism.

### Role of H_2_S in oxygen signalling

The above observations raise the question as to whether endogenous production of H_2_S could nonetheless play a role in oxygen sensing via inhibition of complex IV. Such a hypothesis would be compatible with other current theories regarding oxygen sensing, metabolic signalling and ion channel regulation in type 1 cells [48, 55]. It would also offer an alternative to cytochrome oxidase as the “oxygen sensor” in the form of sulphur dioxygenase. Serious concerns have however been expressed regarding levels of exogenous H_2_S that appear to be required to excite chemoreceptors and whether similar levels could realistically be generated endogenously in vivo [17]. The same considerations must be applied to this study. In order to generate any reproducible [Ca^2+^]_i_ response, H_2_S needed to be > = 2.5 μM; to generate a [Ca^2+^]_i_ response comparable to that seen with hypoxia (Fig. 1) required > = 7.5 μM H_2_S. The need for such high levels of H_2_S to evoke even a small response represents a serious problem for the hypothesis that oxygen sensing is mediated via endogenous H_2_S production. H_2_S is highly membrane permeable (diffusion coefficient = 0.5 cm/s [33]), isolated type 1 cells are small (approximately 5 μm radius) and experiments are conducted in a fast flowing stream of saline which would prevent external H_2_S accumulation. Under these conditions, an internal concentration of just 1 μM H_2_S would drive a transmembrane H_2_S efflux of 1.6 fM/s (calculated using Fick’s first law of diffusion). For a type 1 cell to maintain an intracellular concentration of 1 μM, it would therefore need to continually generate H_2_S at the same rate. For a 5-μm radius cell, this would require H_2_S synthesis at a rate equivalent to 180 mM/min/l intracellular fluid. Estimates of the maximum capacity for tissues to generate H_2_S vary. Liver homogenate (which appears to have one of the highest capacities for H_2_S generation) can generate up to 1 mM/min/kg tissue at saturating concentrations of cysteine/homocysteine [23], but with the use of physiologically relevant concentrations of substrate, this falls to only 8 μM/min/kg liver tissue [49]. These rates are over two orders of magnitude less than that required to sustain a 1-μM concentration gradient of H_2_S across the membrane. The problem is not limited just to a consideration of the kinetics of H_2_S production. There is an even more serious issue regarding the availability of substrate from which to synthesise H_2_S. Isolated type 1 cells can continue to respond to hypoxia for an hour or more whilst being maintained in just a simple saline. To continue to produce H_2_S at the above rate for an hour would require a source of cysteine/homocysteine equivalent to 11 moles/l intracellular fluid. In contrast, tissue levels of homocysteine/cysteine are only in the region of 10–1,000 μM [49]. In conclusion, if the reported values for lipid bilayer permeability to H_2_S are correct, it is highly questionable whether endogenous H_2_S could act as a freely diffusible signalling molecule operating at the micromolar level under the conditions typically used for studying oxygen sensing in isolated cells.

### Mitochondria and oxygen sensing

Finally, it should be noted that this study shows that yet another inhibitor of mitochondrial function mimics the effects of hypoxia in the type 1 cell. One of the most remarkable features of the carotid body is its extraordinary sensitivity to these agents and the fact that mitochondrial function in type 1 cells appears to be exceptionally sensitive to even moderate hypoxia [11, 12]. It is tempting therefore to speculate that, even if H_2_S does not act as a specific messenger in oxygen sensing, physiological levels of H_2_S production together with other endogenous inhibitors of cytochrome oxidase, e.g. NO and CO, might in some way contribute to the unusual oxygen sensitivity of mitochondrial function in these cells.

### Summary

In summary, the following conclusions may be reached:

1. H_2_S excites carotid body type 1 cells via inhibition of background K-channels depolarisation and voltage-gated Ca entry. These effects are qualitatively similar to those of hypoxia.
2. Background K channel inhibition by H_2_S is probably secondary to inhibition of electron transport and oxidative phosphorylation.
3. There was no evidence for any effect of H_2_S on type 1 cell [Ca^2+^]_i_ at levels less than those required to inhibit mitochondrial function nor was there any effect of H_2_S on channel activity in the presence of cyanide. This would appear to rule out a significant role for any additional non-mitochondrial H_2_S receptor-mediated signalling pathway.
4. The levels of H_2_S required to excite type 1 cells via complex IV inhibition make this pathway an unlikely candidate for mediating the effects of hypoxia in isolated cells. A minor role for lower levels of H_2_S in modulating mitochondrial O_2_ sensitivity cannot however be ruled out at this stage.
